# Profiling of bacterial bloodstream infections in hematological and oncological patients based on a comparative survival analysis

**DOI:** 10.1007/s00277-021-04541-9

**Published:** 2021-05-03

**Authors:** Sarah Weber, Aaron Magh, Michael Hogardt, Volkhard A. J. Kempf, Maria J. G. T. Vehreschild, Hubert Serve, Sebastian Scheich, Björn Steffen

**Affiliations:** 1 Department of Medicine, Hematology/Oncology, University Hospital Frankfurt, Frankfurt am Main, Germany; 2 University Center for Infectious Diseases, University Hospital Frankfurt, Frankfurt, Germany; 3 Institute of Medical Microbiology and Infection Control, University Hospital Frankfurt, Frankfurt, Germany; 4 University Center of Competence for Infection Control, Frankfurt, State of Hesse Germany; 5 Department of Medicine, Infectious Diseases Unit, University Hospital Frankfurt, Frankfurt, Germany; 6 German Center for Infection Research (DZIF), Bonn-Cologne, Germany

**Keywords:** Bloodstream infection, Hematology, Oncology, Cancer, Cluster

## Abstract

Bloodstream infections (BSI) are a frequent complication in patients with hematological and oncological diseases. However, the impact of different bacterial species causing BSI and of multiple BSI remains incompletely understood. We performed a retrospective study profiling 637 bacterial BSI episodes in hematological and oncological patients. Based on the 30-day (30d) overall survival (OS), we analyzed different types of multiple BSI and grouped BSI-associated bacteria into clusters followed by further assessment of clinical and infection-related characteristics. We discovered that polymicrobial BSI (different organisms on the first day of a BSI episode) and sequential BSI (another BSI before the respective BSI episode) were associated with a worse 30d OS. Different bacterial groups could be classified into three BSI outcome clusters based on 30d OS: favorable (FAV) including mainly common skin contaminants, *Escherichia* spp. and *Streptococcus* spp.; intermediate (INT) including mainly *Enterococcus* spp., vancomycin-resistant *Enterococcus* spp., and multidrug-resistant gram-negative bacteria (MDRGN); and adverse (ADV) including MDRGN with an additional carbapenem-resistance (MDRGN+CR). A polymicrobial or sequential BSI especially influenced the outcome in the combination of two INT cluster BSI. The presence of a polymicrobial BSI and the assignment into the BSI outcome clusters were identified as independent risk factors for 30d mortality in a Cox multivariate regression analysis. The assignment to a BSI outcome cluster and the differentiated perspective of multiple BSI open new insights into the prognosis of patients with BSI and should be further validated in other patient cohorts.

**Supplementary Information:**

The online version contains supplementary material available at 10.1007/s00277-021-04541-9.

## Introduction

Bloodstream infections (BSI) are a frequent cause of morbidity and mortality in hematological and oncological patients [1, 2] due to a compromised immune system by the underlying disease itself or the respective treatment [3, 4]. Furthermore, these patients are frequently hospitalized and exposed to paths of bacterial transmission as the use of central venous catheters [5].

Frequently, not only one but multiple bacterial organisms are detected causing BSI at the same time or in sequential order. However, data regarding multiple BSI are limited and consistent definitions for multiple BSI are missing [6–8]. Moreover, the role of certain bacterial species on the clinical outcome is not fully unraveled and an early treatment with an effective antibiotic agent is essential for the clinical outcome. In line with this, we and others reported that BSI with multidrug-resistant gram-negative bacteria (MDRGN) or vancomycin-resistant *Enterococcus* spp. (VRE) are associated with an increased mortality in hematological and oncological patients [2, 9–12]. However, contradictory data exist as well [13, 14]. Besides, the evaluation of a BSI with detection of potential skin contaminants is challenging [15] and the impact of BSI with uncommon bacterial species is only sparsely determined. Therefore, comparative survival analyses and characterizations of BSI caused by different bacterial organisms are highly needed.

We elucidate in this study the impact of multiple BSI and report a comprehensive analysis of different BSI-associated bacteria based on a 30-day survival classification system.

## Methods

### Study design

This retrospective, single-center study includes 637 bacterial BSI episodes detected 09/2006–06/2019 in hematological or oncological patients admitted to our department. We examined the impact of multiple BSI detections with 30-day (30d) overall survival (OS) being the primary endpoint. Moreover, clinical relevance of BSI-associated bacteria was analyzed by clustering them into groups based on 30d mortality. The study was approved by the local ethical committee (approval: SHN-10-2017). The analysis includes the revision of BSI episodes with enterococci and gram-negative bacteria that were already examined in earlier publications in a different context [9, 10].

### Microbiological testing and definitions

In the event of fever or other signs of systemic infection, blood cultures were taken at the discretion of the treating physicians. Subsequent detection of at least one bacterial organism in one blood culture was defined as BSI. In case of common skin contaminants (CSC) such as coagulase-negative *Staphylococcus* spp., *Bacillus* spp., *Corynebacterium* spp.*, Cutibacterium* spp., and *Micrococcus* spp., two positive blood cultures within 48 h were needed to fulfill the BSI definition. MDRGN was defined as *Enterobacteriaceae*, *Acinetobacter baumannii*, or *Pseudomonas aeruginosa* with resistance against at least three out of four antibiotic classes as described previously [9]. In case of an additional carbapenem resistance, the bacteria were defined as MDRGN+CR (resistant against all four antibiotic classes*).* MDRGN, MDRGN+CR, VRE, and methicillin-resistant *Staphylococcus aureus* (MRSA) were collectively termed multidrug-resistant organisms (MDRO).

Detected bacterial species were grouped together for subsequent analysis depending on their genera and antibiotic resistance profiles. Rare organisms were further summarized depending on their bacterial family, anaerobic growth behavior, and gram staining.

A 30d period with single or repeated detection of the same bacterial organism was termed as a BSI episode. Multiple BSI were further subclassified: Repeated detection of the same bacterial organism was termed as repeatedly detected BSI. A polymicrobial BSI was defined by the detection of different bacterial organisms on the first day of a BSI episode. The detection of a different bacterial organism on days 2–30 of a BSI episode indicated an overlapping BSI. A sequential BSI was defined by the detection of another BSI any time before the respective BSI episode. Polymicrobial episodes were counted separately for all analyses except 30d OS analyses of polymicrobial, overlapping and sequential BSI, where concurrent polymicrobial episodes were included only once to avoid data distortion. All microbiological analyses were performed as described previously [16].

### Treatment with antibiotic agents

An antimicrobial prophylaxis with levofloxacin was routinely administered to patients with an estimated prolonged neutropenia (≥ 10 days). Patients undergoing hematopoietic stem cell transplantation (HSCT), patients with acute lymphoblastic leukemia (ALL), lymphomas, or other reasons increasing the risk for a *Pneumocystis jirovecii* pneumonia routinely received a prophylaxis with cotrimoxazole/trimethoprim. During HSCT, patients also routinely received a prophylaxis with levofloxacin or cefotaxime, a broad-spectrum azole and acyclovir. If patients presented with clinical signs of infection, an empiric antibiotic therapy was initiated: Neutropenic patients routinely received piperacillin/tazobactam, whereas patients with a known MDRGN colonization received imipenem or meropenem. After identification of a microorganism in patient samples, the antibiotic treatment was adjusted accordingly if necessary.

### Statistical analysis

Differences of nominal variables were evaluated using chi-square or Fischer’s exact test (post hoc test: Bonferroni-correction). Metric variables were compared using the Mann-Whitney-*U* or Kruskal-Wallis test (post hoc test: Dunn-Bonferroni). Estimation of 30d OS was calculated using the Kaplan-Meier method with comparison of the groups via log rank test. Factors that might independently contribute to 30d mortality were tested in a simple Cox analysis and variables with a *p*-value <0.1 were included into a multivariate analysis. All statistical tests were two-tailed and considered to be significant with *p*<0.05. If data were not available, BSI episodes were excluded from the respective analysis. For classification of bacterial groups based on 30d OS, we performed a hierarchical cluster analysis using Ward’s method of minimal variance and squared Euclidean distance leading to different BSI outcome clusters. Comparison of characteristics and the Cox regression analysis were performed with SPSS (version 25.0; IBM). R (version 3.5.0) was used for the 30d OS and the cluster analysis.

## Results

**Fig. 1:**
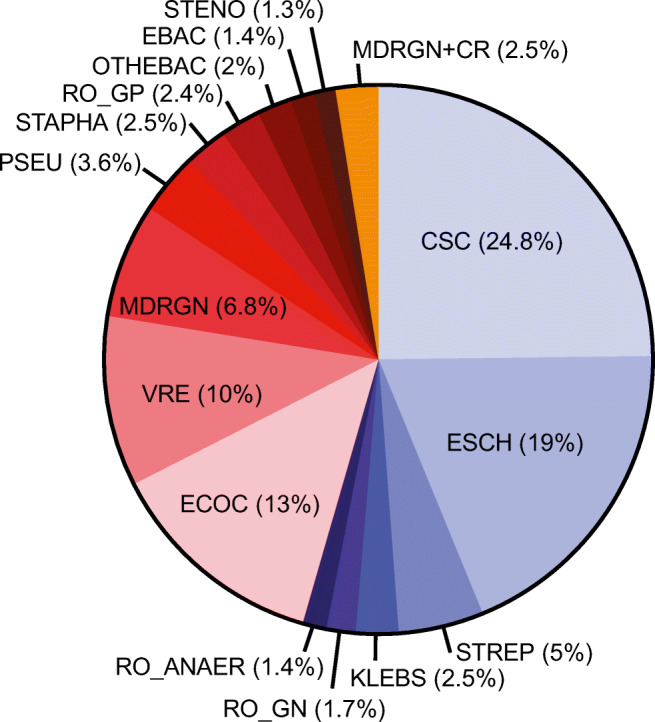
Pie chart showing the frequency of BSI due to different bacterial groups (*n*= 637 BSI episodes). CSC, common skin contaminants; EBAC, *Enterobacter* spp.; ECOC, *Enterococcus* spp.; ESCH, *Escherichia* spp. (only represented by the species *E. coli*) *Klebsiella* spp.; MDRGN, multi-resistant gram-negative bacteria; MDRGN+CR, MDRGN with additional carbapenem resistance; OTHEBAC, other *Enterobacterales*; PSEU, *Pseudomonas* spp.; RO_AN, anaerobic bacteria (rare organisms); RO_GN, gram-negative rare organisms; RO_GP, gram-positive rare organisms; STAPHA, *S. aureus*; STENO, *Stenotrophomonas* spp. (only represented by the species *S. maltophilia*); STREP, *Streptococcus* spp.; VRE, vancomycin-resistant enterococci

The aim of this study was to elucidate characteristics of bacterial BSI with an impact on mortality. Overall, we analyzed 637 bacterial BSI episodes in 391 hematological or oncological in-patients. Most of the patients suffered from malignant hematological diseases (*n*=359, 91.8%), a small proportion presented with other hematological disorders (*n*=14, 3.6%), or solid tumors (*n*=18, 4.6%) (Table S1). Of all patients, 154 (39.4%) showed at least 2 up to 10 BSI episodes during the study period. Overall 30d OS was 87.7% (95% confidence interval (CI) 85.1–90.3) with no differences between gram-negative and gram-positive BSI episodes (Figure S1). The majority of detected BSI bacteria were found in the CSC group with 24.8%, in the *Escherichia* spp. (ESCH) group with 19.0%, in the *Enterococcus* spp. (ECOC) group with 13.0%, in the VRE group with 10%, in the MDRGN group with 6.8%, and in the *Streptococcus* spp. (STREP) group with 5.0% (Fig. 1, Table S2).

### Impact of multiple BSI on patient outcome

**Fig. 2:**
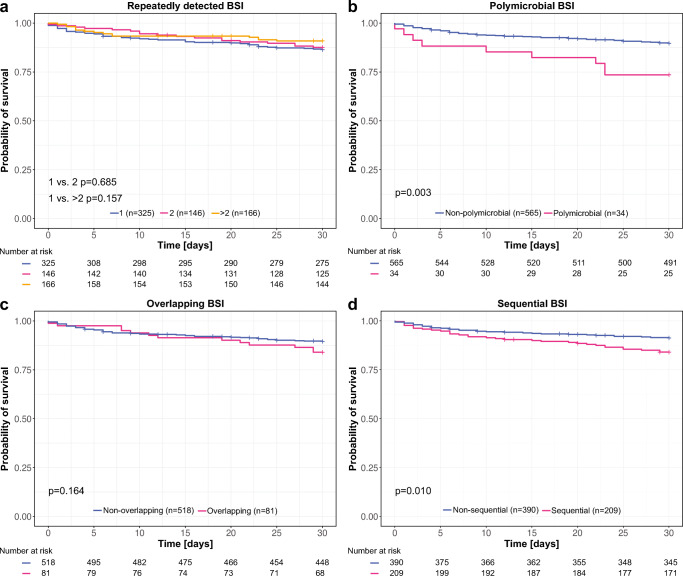
Kaplan-Meier plots showing 30d OS for BSI episodes with and without repeatedly detected BSI (same organism within one BSI episode) (**a**), polymicrobial BSI (different organisms on the first day of a BSI episode) (**b**), overlapping BSI (different organism on days 2–30 of a BSI episode) (**c**), and sequential BSI (another BSI before the respective BSI episode) (**d**)

To assess if multiple BSI and different timepoints of multiple BSI detections impact 30d OS, we differentiated between BSI episodes with repeatedly detected BSI (same organism within one BSI episode), polymicrobial BSI (different organism on the first day of a BSI episode), overlapping BSI (different organism on days 2–30 of a BSI episode), and sequential BSI (another BSI before the respective BSI episode). Regarding repeatedly detected BSI, no 30d OS differences were observed: In 325 BSI episodes with singular detection, the 30d OS was 86.4% (95% CI 82.7–90.2), in 146 BSI episodes with twice detected organisms, 87.6% (95% CI 82.7–90.2), and in 166 BSI episodes with 3–10 times detected organisms, 90.9% (95% CI 86.5–95.4) (Fig. 2a). Polymicrobial BSI occurred in 5.7% of the cases with 2–4 organisms in parallel. Thereby, polymicrobial BSI showed a worse 30d OS with 73.5% (95% CI 60.1–90.0) compared to non-polymicrobial BSI with 89.6% (95% CI 87.1–92.2) (*p*=0.003) (Fig. 2b). Overlapping BSI were found in 13.5% of the cases without a difference comparing the 30d OS of overlapping BSI (84.0%; 95% CI 76.3–92.3) to non-overlapping BSI (89.5%; 95% CI 86.9–92.2) (Fig. 2c). Still, no differences were found when including only overlapping BSI within the first 5 days after BSI onset (Figure S2). A sequential BSI was detected in 34.9% of the cases. 30d OS was worse in case of a sequential BSI with 84.1% (95% CI 79.3–89.2) compared to a non-sequential BSI with 91.2% (95% CI 88.4–94.1) (*p*=0.010) (Fig. 2d).

### Clustering of different BSI bacterial groups based on 30d OS

**Fig. 3:**
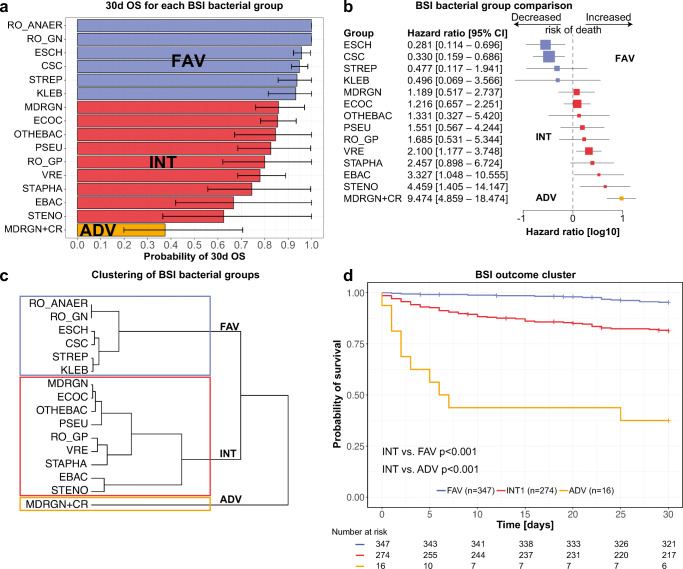
Bar chart showing 30d OS and 95% confidence intervals for BSI of different bacterial groups (**a**). Forest plot depicting hazard ratios for 30d mortality and 95% confidence intervals comparing each BSI group to all others showing all BSI groups with at least one event of death (**b**). Dendrogram clustering the BSI bacterial groups depending on their 30d OS into three clusters by hierarchical cluster analysis using Ward’s method (**c**). Kaplan-Meier plot showing 30d OS for BSI episodes according to the classification into three clusters (**d**). Colors indicate the affiliation of the groups to the three clusters

We analyzed the 30d OS of each BSI bacterial group (Table S3, Fig. 3a, Figure S3) and the risk of 30d mortality comparing each BSI bacterial group to all others (Fig. 3b). Next, we performed a hierarchical cluster analysis based on the 30d OS of the different BSI causing pathogens (Fig. 3c). Deducing from this analysis, we assigned the bacterial groups to three BSI outcome clusters: favorable (FAV), intermediate (INT), and adverse (ADV). The corresponding 30d OS of these three clusters were 95.3% (95% CI 93.1–97.6) for the FAV, 81.3% (95% CI 76.8–86.0) for the INT, and 37.5% (95% CI 19.9–70.6) for the ADV cluster (Fig. 3d).

Patient characteristics for the three BSI outcome clusters are listed in Table 1a and Table S4. Median age was ascending over the clusters from 52 (19–82) in the FAV to 60.5 (32–74) in the ADV cluster (*p*=0.001). Moreover, the clusters differed in the number of prior lines of therapy (*p*=0.018, more first-line therapies in FAV with 51.3% and less in INT with 38.0%), the number of patients in the phase of HSCT (*p*<0.001, less in FAV with 26.2% and higher in INT with 40.5%) and the administration of an immunosuppressive therapy at the time of BSI detection (*p*=0.003, lower in FAV with 25.6% and higher in INT with 38.3%). The prevalence of severe neutropenia increased slightly from the FAV with 69.2% to the ADV cluster with 87.5%, although not being significant (*p*=0.057). The differences became even more apparent for the duration of severe neutropenia before the BSI with a median of 6 (0–204) days in the FAV, 11 (0–204) days in the INT, and 17.5 (0–100) days in the ADV cluster (*p*<0.001). Levels of C-reactive protein (CRP) increased from 5.1 mg/dl (0.0–50.7) in the FAV to 11.9 mg/dl (0.2–48.2) in the ADV cluster (*p*=0.001). On the contrary, albumin level decreased from 3.4 g/dl (1.7–4.8) in the FAV to 3.1 g/dl (1.7–3.8) in the ADV cluster (*p*<0.001).

**Table 1:** Comparison of patient characteristics between three BSI bacteria clusters (FAV, INT, ADV). Unless otherwise specified, variable values refer to day 1 of a BSI episode. The phase of HSCT was defined from start of the conditioning regimen until 100 days after transplantation. The Charlson comorbidity index was used to assess the overall comorbidity burden using the updated Charlson comorbidity index with a score of at least three defining high risk [17, 18]. The common terminology criteria for adverse events (CTCAE) were used to grade mucositis [19]. Severe neutropenia was defined as an absolute neutrophil count of less than 500/μl. *p*-values < 0.005 indicate differences between the three clusters. After a significant Kruskal-Wallis test, we used the Dunn-Bonferroni post hoc test to assign pairwise significant differences (^†^/^‡^). After a significant chi-square test, significant differences were assigned via post hoc test using the Bonferroni-correction (*). ***HCT-CI*, hematopoietic cell transplantation-specific comorbidity index;** ***ICU*, intensive care unit;** ***N/A*, not available**

	All BSI (*n*=637)	FAV (*n*=347)	INT (*n*=274)	ADV (*n*=16)	*p*-value
Age, median (range)	54 (17–87)	52 (19–82)^†^	56 (17–87)^†^	60.5 (32–74)	**0.003**
Female sex, *n* (%)	235 (36.9)	129 (37.2)	102 (37.2)	4 (25.0)	0.607
Phase of disease, *n* (%)					0.366
Newly diagnosed/under first line therapy	331 (52.0)	190 (54.8)	134 (48.9)	7 (43.8)	
In complete remission	56 (8.8)	33 (9.5)	22 (8.0)	1 (6.3)	
Refractory disease/relapse	250 (39.2)	124 (35.7)	118 (43.1)	8 (50.0)	
Curative treatment approach, *n* (%)	538 (84.5)	298 (85.9)	229 (83.6)	11 (68.8)	0.157
Number of therapy lines, *n* (%)					**0.018**
First line	288 (45.2)	178 (51.3)*	104 (38.0)*	6 (37.5)	
Second line	200 (31.4)	95 (27.4)	100 (36.5)	5 (31.3)	
Further line	149 (23.4)	74 (21.3)	70 (25.5)	5 (31.3)	
In the phase of HSCT, *n* (%)	207 (32.5)	91 (26.2)*	111 (40.5)*	5 (31.3)	**<0.001**
HSCT in the past, *n* (%)	130 (20.4)	77 (22.2)	49 (17.9)	4 (25.0)	0.375
Comorbidities, *n* (%)					
Diabetes mellitus	88 (13.8)	50 (14.4)	36 (13.1)	2 (12.5)	0.891
Heart disease	139 (21.8)	77 (22.2)	56 (20.4)	6 (37.5)	0.267
Liver disease	43 (6.8)	18 (5.2)	25 (9.1)	0 (0.0)	0.084
Lung disease	54 (8.5)	31 (8.9)	21 (7.7)	2 (12.5)	0.719
Renal failure	70 (11.0)	44 (12.7)	26 (9.5)	0 (0.0)	0.164
Charlson comorbidity index, median (range)	2 (0–10)	2 (0–10)	2 (0–10)	2 (2–6)	0.484
Hospital admission within previous 90 days, *n* (%)	491 (77.1)	263 (75.8)	215 (78.5)	13 (81.3)	0.667
ICU admission within previous 90 days, *n* (%)	40 (6.3)	20 (5.8)	19 (6.9)	1 (6.3)	0.837
Indwelling central catheter, *n* (%)	522 (81.9)	275 (79.3)	232 (84.7)	15 (93.8)	0.101
Parenteral nutrition, *n* (%)	84 (13.2)	40 (11.5)	43 (15.7)	1 (6.3)	0.222
Immunosuppressive therapy, *n* (%)	199 (31.2)	89 (25.6)*	105 (38.3)*	5 (31.2)	**0.003**
Corticosteroids within previous 7 days, *n* (%)	260 (42.7)(N/A=28)	145 (43.8)(N/A=16)	107 (40.7)(N/A=11)	8 (53.3)(N/A=1)	0.523
Chemotherapy within previous 30 days, *n* (%)	502 (78.8)	264 (76.1)	224 (81.8)	14 (87.5)	0.158
Severe (grade 3/4) mucositis at BSI, *n* (%)	72 (11.3)	36 (10.4)	34 (12.4)	2 (12.5)	0.721
Severe neutropenia, *n* (%)	463 (72.7)	240 (69.2)	209 (76.3)	14 (87.5)	0.057
Duration of severe neutropenia before BSI (days), median (range)	7 (0–204)	6 (0–204)^†^	11 (0–204)^†^	17.5 (0–100)	**<0.001**
Creatinine (mg/dl), median (range)	0.8 (0.2–7.4)	0.8 (0.3–5.1)	0.8 (0.2–7.4)	1.2 (0.4–2.4)	0.159
CRP (mg/dl), median (range)	6.3 (0.0–50.7)	5.1 (0.0–50.7)^†,‡^	7.4 (0.1–49.5)^†^	11.9 (0.2–48.2)^‡^	**0.001**
Albumin (g/dl), median (range)	3.3 (1.5–4.8)(N/A=20)	3.4 (1.7–4.8)(N/A=9)^†^	3.2 (1.5–4.7)(N/A=11)^†^	3.1(1.7–3.8)	**<0.001**

Significant values (*p*-value <0.05) are marked in bold letters to highlight statistical significance

Infection- and outcome-related characteristics are shown in Table 2. Considering all BSI episodes, differences were found in the distribution of polymicrobial (*p*=0.003), overlapping (*p*=0.009) and sequential BSI (*p*=0.003) with a lower frequency in the FAV and a higher frequency in the INT cluster for all of them. As polymicrobial and sequential BSI showed a worse 30d OS over all clusters, we compared the 30d OS for different combinations of BSI outcome clusters and the corresponding non-polymicrobial or non-sequential BSI stratified for each cluster (Figure S4). Here, a difference in the 30d OS was found for the combination of at least two in parallel detected INT cluster BSI compared to non-polymicrobial INT BSI (*p*<0.001) and for sequential BSI with an INT cluster BSI following another INT cluster BSI compared to non-sequential INT cluster BSI (*p*=0.011), whereas no differences were found for other combinations especially not including FAV cluster BSI. Nosocomial acquisition of the BSI differed among the clusters (*p*<0.001) and was rarer in the FAV and more frequent in the INT cluster. The proportion of BSI episodes receiving an adequate empiric antibiotic therapy decreased from 82.6% for the FAV to 28.6% for the ADV cluster (*p*<0.001). Furthermore, the proportion of prior colonization with an MDRO and the proportion of microbiological evidence of a non-BSI bacterial infection +/−30d around the first BSI detection increased with every more adverse cluster (*p*<0.001). The proportion of infections with the BSI-associated organism 30d before BSI onset was equally distributed among the clusters. The number of ICU admissions and especially the number of ICU admissions due to infectious diseases during the 30d follow-up period differed between the clusters (*p*<0.001) with the latter increasing from 7.8% in the FAV to 37.5% in the ADV cluster.

**Table 2:** Comparison of **infection- and outcome-related characteristics between three BSI bacteria clusters (FAV, INT, ADV).** Nosocomial acquisition of the BSI was assumed, when at least 72 h elapsed between hospital admission and BSI detection. An empiric antibiotic therapy usually given within the first 48 h was defined as adequate or inadequate depending on the antibiotic susceptibility testing or, if data were not available, according to the EUCAST criteria and expert rules [20]. *p*-values < 0.005 indicate differences between the three clusters. After a significant chi-square test, significant differences were assigned via post hoc test using the Bonferroni-correction (*). ***ICU*, intensive care unit;** *MDRO*, multidrug-resistant organism

	All BSI(*n*=637)	FAV(*n*=347)	INT(*n*=274)	ADV(*n*=16)	*p*-value
Polymicrobial BSI	72 (11.3)	28 (8.1)*	44 (16.1)*	0 (0.0)	**0.003**
Overlapping BSI	84 (13.2)	33 (9.5)*	49 (17.9)*	2 (12.5)	**0.009**
Sequential BSI	226 (35.5)	103 (29.7)*	115 (42.0)*	8 (50.0)	**0.003**
Nosocomial acquisition of BSI, *n* (%)	480 (75.4)	240 (69.2)*	226 (82.5)*	14 (87.5)	**<0.001**
Adequate empiric antibiotic therapy, *n* (%)	415 (75.6)(N/A=88)	242 (82.6)(N/A=54)*	169 (69.8)(N/A=32)*	4 (28.6) (N/A=2)*	**<0.001**
Prior colonization with any MDRO, *n* (%)	344 (54.0)	162 (46.7)*	168 (61.3)*	14 (87.5)*	**<0.001**
Prior colonization with BSI-associated MDRO referring to the affected BSI groups, *n* (% of group)			MDRGN: 30/43 (69.8), VRE: 37/64 (57.9)	MDRGN+CR: 9/16 (56.3)	
Microbiological evidence of a non-BSI bacterial infection +/− 30 days, *n* (%)	164 (25.7)	71 (20.5)*	83 (30.3)	10 (62.5)*	**<0.001**
Infection with BSI associated organism within previous 30 days, *n* (% of bacterial infections)	32/164 (19.5)	15/71 (21.1)	16/83 (19.3)	1/10 (10.0)	0.706
ICU admission during 30-day follow-up period, *n* (%)	91 (14.3)	34 (9.8)*	51 (18.6)*	6 (37.5)*	**<0.001**
ICU admission due to infectious diseases during 30-day follow-up period, *n* (%)	72 (11.3)	27 (7.8)*	39 (14.2)	6 (37.5)*	**<0.001**
Cause of death: Infectious disease, *n* (% of deaths)	46/77 (59.7)	9/16 (56.3)	29/51 (56.9)	8/10 (80.0)	0.375

Significant values (*p*-value <0.05) are marked in bold letters to highlight statistical significance

To examine, if the differences concerning polymicrobial and sequential BSI as well as the different clusters were still existent in the presence of other risk factors for 30d mortality, we performed a multivariate Cox regression analysis (Table 3). Univariate analysis identified age ≥ 60 years, a Charlson comorbidity index ≥ 3, second or higher therapy line, a polymicrobial BSI, a sequential BSI, and the classification into different BSI outcome clusters as potential risk factors for 30-day mortality. Of these, receipt of the second or higher therapy line (HR 1.809 (95% CI 1.028–3.181), *p*=0.040), an adequate empiric antibiotic therapy (HR 0.547 (95% CI 0.339–0.972), *p*=0.039), a polymicrobial BSI (HR 2.682 (95% CI 1.448–4.968), *p*=0.002), and the classification into different BSI outcome clusters (INT compared to FAV: HR 3.958 (95% CI 2.069–7.570), *p*<0.001; ADV compared to INT: HR 6.367 (95% CI 2.937–13.801), *p*<0.001) were identified as independent risk factors for 30-day mortality in the multivariate analysis.

**Table 3:** Simple and multivariate regression analysis of risk factors for 30-day mortality including the BSI outcome cluster. For the clusters, three dichotomic variables comparing one cluster with the next adverse cluster were generated and included into the analysis. All factors with *p*<0.1 in the simple Cox regression analysis were considered for the multivariate Cox regression analysis. For empiric antibiotic therapy, some BSI episodes could not be assigned because antibiotic susceptibility data could not be accessed. The analysis still represents 86.2% of all BSI episodes. *CI*, confidence interval; ***N/A*, not available**

Characteristics	Descriptive (*n*, %)		Simple regression			Multivariate regression		
	Non-survivors (30 days) (*n*= 77)	Survivors (30 days) (*n*= 560)	HR	95% CI	*p*-value	HR	95% CI	*p*-value
Age ≥ 60 years	37 (48.1)	192 (34.3)	1.743	1.115 –2.726	**0.016**	1.427	0.862–2.360	0.116
Female sex	50 (64.9)	352 (62.9)	1.086	0.680–1.734	0.731			
Charlson comorbidity index ≥ 3	28 (36.4)	135 (24.1)	1.709	1.074–2.719	**0.024**	1.677	0.985–2.856	0.057
Therapy line ≥ 2	20 (26.0)	268 (47.9)	2.461	1.479–4.096	**0.001**	1.809	1.028–3.181	**0.040**
Nosocomial BSI	59 (76.6)	421 (75.2)	1.081	0.638–1.832	0.754			
No adequate empiric antibiotic therapy	39 (58.2)	376 (78.0)	2.425	1.492–3.941	**0.000**	1.867	1.120–3.111	**0.017**
Polymicrobial BSI	19 (24.7)	53 (9.5)	2.772	1.651–4.654	**<0.001**	2.676	1.445–4.954	**0.002**
Sequential BSI	41 (53.2)	184 (32.9)	2.157	1.379–3.375	**<0.001**	1.468	0.871–2.474	**0.150**
BSI cluster								
FAV	16 (20.8)	331 (59.1)						
INT (compared to FAV)	51 (66.2)	223 (39.8)	4.402	2.510–7.719	**<0.001**	7.874	3.649–16.996	**<0.001**
ADV (compared to INT)	10 (13.0)	6 (1.1)	5.249	2.658–10.366	**<0.001**	3.732	1.946–7.160	**<0.001**

Significant values (*p*-value <0.05) are marked in bold letters to highlight statistical significance

## Discussion

In this study, we assessed the impact of multiple BSI and of different detected bacterial pathogens on patient outcome based on a comparative survival analysis. To the best of our knowledge, this is the first study describing detailed outcome comparisons and clustering of different BSI bacterial groups in hematological and oncological patients in a relatively large dataset. The overall mortality of all BSI was 12.3%. In a large Japanese study in hospitalized patients, 15.2% died after 30 days [21]. However, in a smaller study from Mexico including only cancer patients, the 30-day mortality was 22% [2]. In both reported studies, the three most common bacterial species were *E. coli, S. aureus*, and *Klebsiella* spp.*,* whereas we describe a BSI cohort consisting mainly of CSC, *E. coli*, and *Enterococcus* spp*.*, which might explain the observed differences.

Investigating the role of multiple BSI, we uncovered that repeatedly detected BSI (same organism within one BSI episode) and overlapping BSI (different organism on days 2–30 of a BSI episode) did not affect early mortality. However, the definitions of multiple BSI are inconsistent across the literature. Pavlaki et al. defined polymicrobial BSI by the detection of different pathogens from one pair of culture bottles and observed higher mortality rates [22], but their study was not restricted to cancer patients. Royo-Cebrecos et al. have shown that polymicrobial BSI are also associated with a worse 30d OS in cancer patients defining polymicrobial as the detection of 2 organisms within 72 h [6]. In our analysis, polymicrobial BSI defined as the detection of different organisms on the first day of a BSI episode had a negative impact on 30-day OS (73.5% vs. 89.6%), but 5-day overlapping BSI (different organism on days 2–5 of a BSI episode) showed no lower survival probability. Besides, sequential BSI (another BSI before the respective BSI episode) were associated with a worse 30d OS (84.1% vs. 91.2%). Although BSI recurrence in hematological patients is not well studied, sequential BSI (there named recurrent) were also associated with a higher risk of death in a population-based surveillance study [23].

To compare the prognostic impact of different BSI bacteria on the outcome, we performed a hierarchical cluster analysis based on the 30d OS leading to three different clusters. As expected, *E. coli* and CSC fall into the bacterial groups with a better outcome as described in the literature [24–26] and multidrug-resistant gram-negative bacteria with additional carbapenem resistance (MDRGN+CR) had the comparably highest mortality rate as also observed by others [27, 28]. However, it was surprising that BSI caused by *Enterobacter* spp. were associated with a high risk for 30d mortality. *Enterobacter* spp. have been described previously to contribute to a higher mortality rate ranging from 20 to 25% [29, 30] but here, we show that the mortality rate for *Enterobacter* spp.-associated BSI was at least as high as for MDRGN and VRE. Moreover, we identified *S. maltophilia* BSI as one pathogen with the highest mortality rate. This is in line with previous studies that demonstrated a mortality rate up to 64% [31, 32] and might be explained by its intrinsic multidrug resistance phenotype.

Our three identified clusters showed differences in underlying patient characteristics with a higher prevalence of adverse risk factors (increased age, therapy lines, HSCT, immunosuppressive therapy, duration of neutropenia) in the INT and ADV clusters compared to the FAV cluster. As bacteria were assigned to the clusters based on their outcome, it seems rational that poor prognostic factors indicate a poor prognosis. Vice versa, this shows that the method indeed reflects the distribution of prognostic factors. However, it cannot be discriminated if the different distributions of prognostic factors between different BSI groups are a result of the cluster assignment or might reflect a different vulnerability to infections with bacteria of certain clusters. Therefore, it is all the more interesting that the BSI clusters score even independently of other predictive factors in a multivariate analysis for 30d mortality.

Additionally, we could demonstrate that the negative impact of polymicrobial and sequential BSI relies on the combination of the detected pathogens. The effects were primarily observed for pathogens falling in the INT cluster (mainly *Enterococcus* spp., VRE, MDRGN, and *Pseudomonas* spp.) and not for pathogens falling in the FAV cluster (mainly CSC, *E. coli*, *Streptococcus* spp., and *Klebsiella* spp.). This might indicate that especially BSI caused by pathogens of the INT cluster represent a patient cohort of increased risk for mortality. As sequential BSI was no independent risk factor for 30d mortality in our multivariate analysis in the presence of other factors such as higher therapy lines, this factor might be mainly an indicator for an increased time of hospitalization and a worse general health state.

However, assignment to the clusters as well as the presence of a polymicrobial BSI wereidentified as risk factors for 30d mortality in a multivariate analysis independently of other risk factors as age, pre-existing medical conditions, and adequateempiric antibiotic therapy. Given the fact that an inadequate empiric antibiotic therapy is a major risk factor for a BSI-related death [33, 34] and that BSI caused by MDRO are more likely to be treated inadequately [35, 36], it was surprising that a more adverse cluster was associated with an adverse outcome even independently of the empiric therapy. Additional studies may further unravel if the detected pathogen associated with a certain outcome clusters is rather a more precise indicator for a worse general health state or cause of the increased mortality itself. Therefore, the role of the pathogen itself beside the resistance profile might be underscored in other studies yet.

## Supplementary Information

Figure S1: Kaplan-Meier plots showing 30d OS for all 637 BSI episodes (A) and for gram-positive compared to gram-negative BSI episodes (B). (PNG 158 kb).

High resolution image (EPS 4770 kb).

Figure S2: Kaplan-Meier plot showing 30d OS for BSI episodes with maximally 5-day overlapping BSI (different organism on day 2–30 of a BSI episode). (PNG 170 kb).

High resolution image (EPS 2493 kb).

Figure S3: Kaplan-Meier plots showing 30d OS for all bacterial organism groups (A) and for EBAC, ESCH, KLEB and OTHEBAC (B), MDRGN, MDRGN+CR, PSEU, RO-AN, RO_GN and STENO (C) as well as CSC, ECOC, RO_GP, STAPHA, STREP and VRE (D) separately. (PNG 426 kb).

High resolution image (EPS 5675 kb).

Figure S4: Kaplan-Meier plots showing 30d OS for polymicrobial BSI episodes depending on the two highest parallel detected clusters and the corresponding non-polymicrobial BSI cluster stratified for the highest cluster present (A+B). Kaplan-Meier plots showing 30d OS for sequential BSI episodes depending on the highest previously detected cluster (first cluster) and the corresponding non-sequential BSI cluster stratified for the cluster of the current BSI episode (second cluster) (C-E). Groups with <5 BSI episodes were not included in the log rank test analysis and were only displayed descriptively. (PNG 493 kb).

High resolution image (EPS 5658 kb).

ESM 1 (DOCX 14 kb).

ESM 2 (DOCX 17 kb).

ESM 3 (DOCX 14 kb).

ESM 4 (DOCX 13 kb).

## Data Availability

Not applicable.
