# Supplementary figures and images for: Profiling of bacterial bloodstream infections in hematological and oncological patients based on a comparative survival analysis
Source: Ann Hematol. 2021 May 3;100(6):1593–602. doi: 10.1007/s00277-021-04541-9 (PMC8116230; doi:10.1007/s00277-021-04541-9)

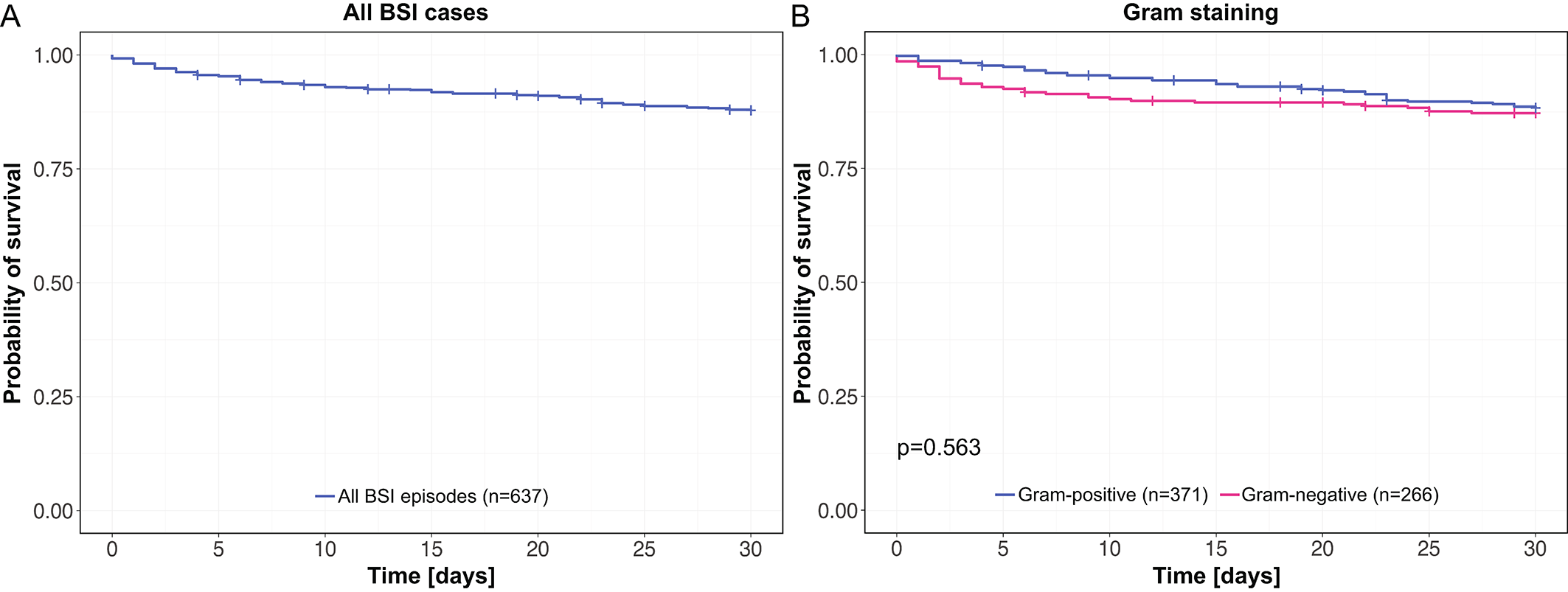

Supplement: Supplementary file 1 — Kaplan-Meier plots showing 30d OS for all 637 BSI episodes (A) and for gram-positive compared to gram-negative BSI episodes (B). (PNG 158 kb). [file 277_2021_4541_Fig4_ESM.png]

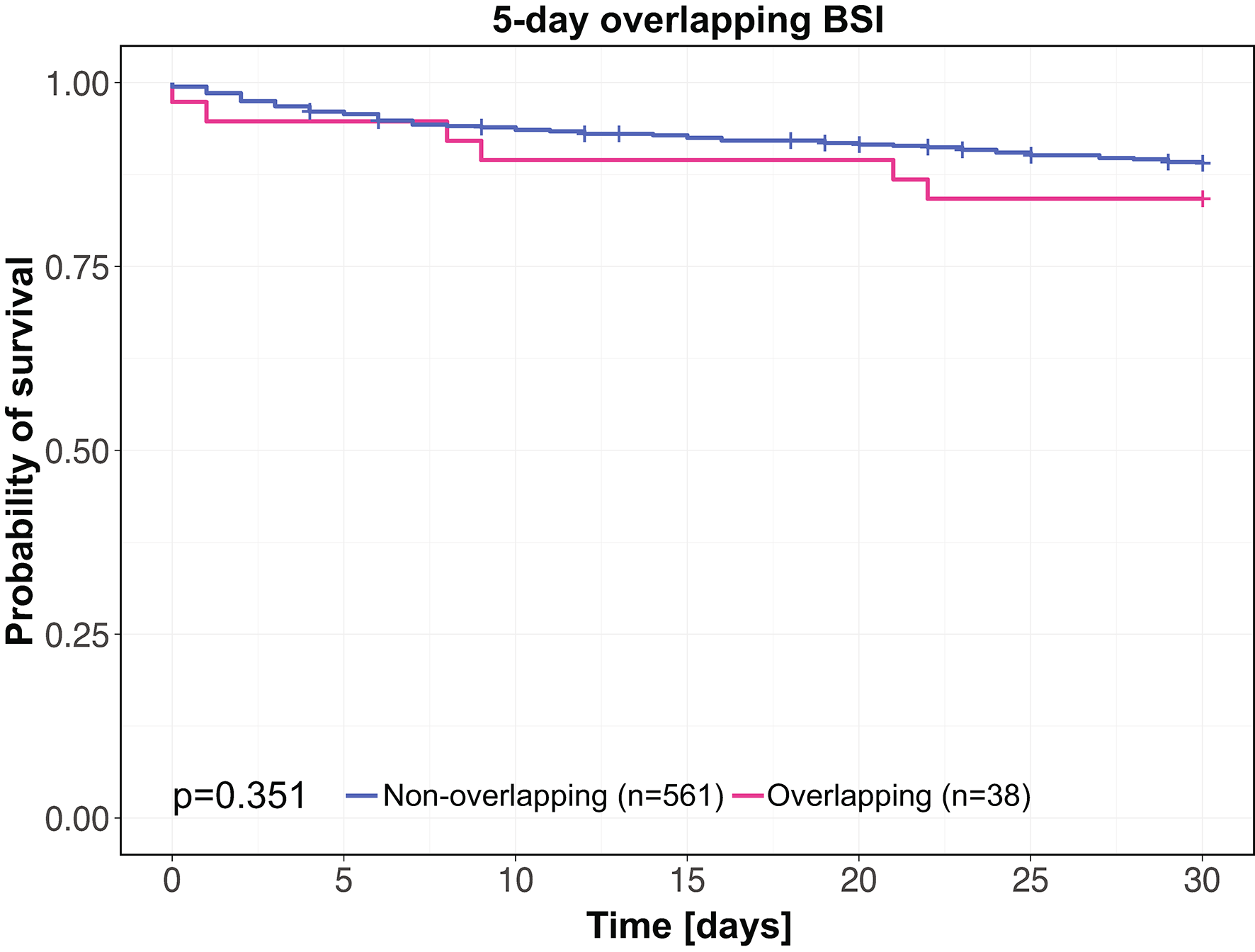

Supplement: Supplementary file 3 — Kaplan-Meier plot showing 30d OS for BSI episodes with maximally 5-day overlapping BSI (different organism on day 2–30 of a BSI episode). (PNG 170 kb). [file 277_2021_4541_Fig5_ESM.png]

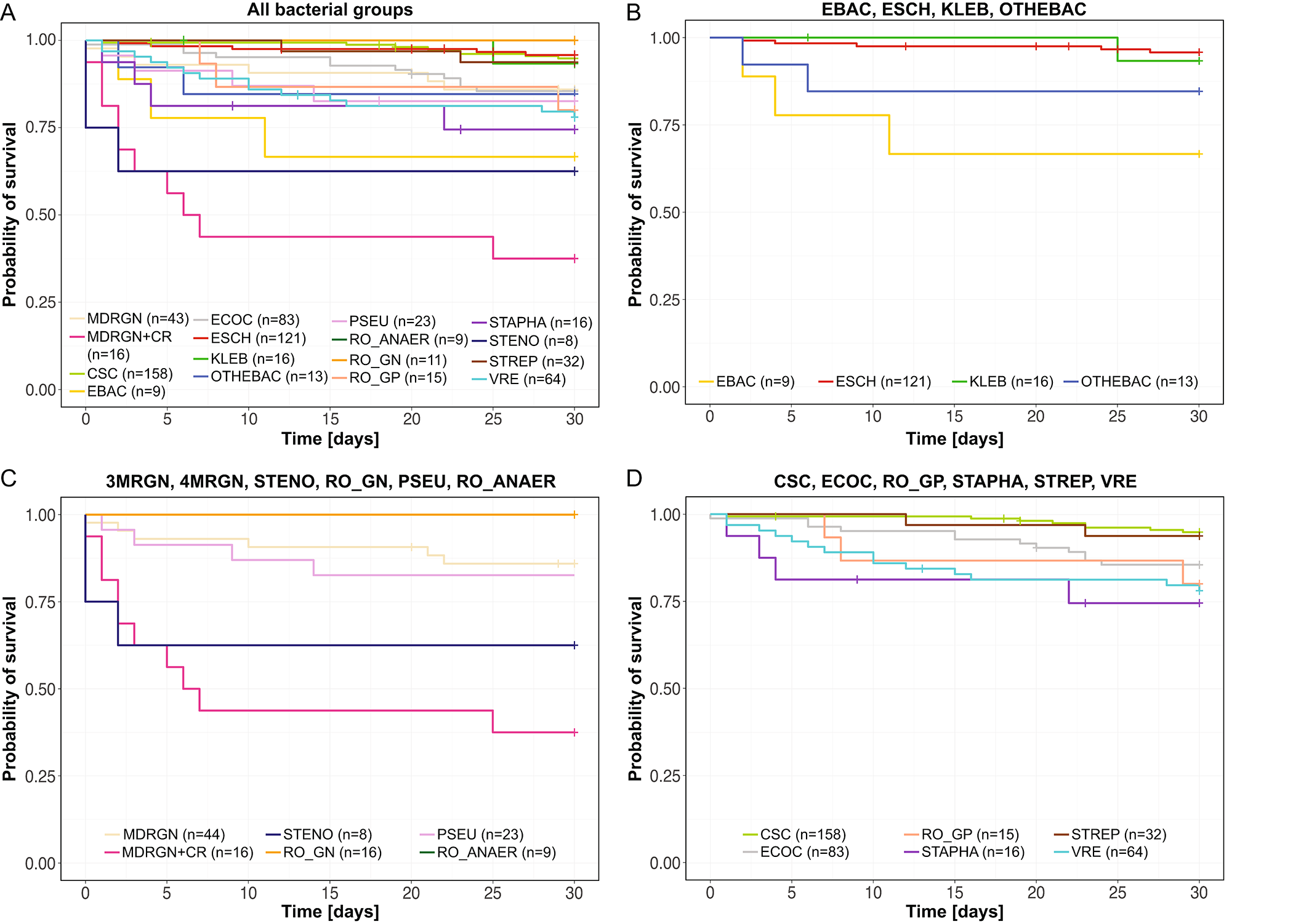

Supplement: Supplementary file 5 — Kaplan-Meier plots showing 30d OS for all bacterial organism groups (A) and for EBAC, ESCH, KLEB and OTHEBAC (B), MDRGN, MDRGN+CR, PSEU, RO-AN, RO_GN and STENO (C) as well as CSC, ECOC, RO_GP, STAPHA, STREP and VRE (D) separately. (PNG 426 kb). [file 277_2021_4541_Fig6_ESM.png]

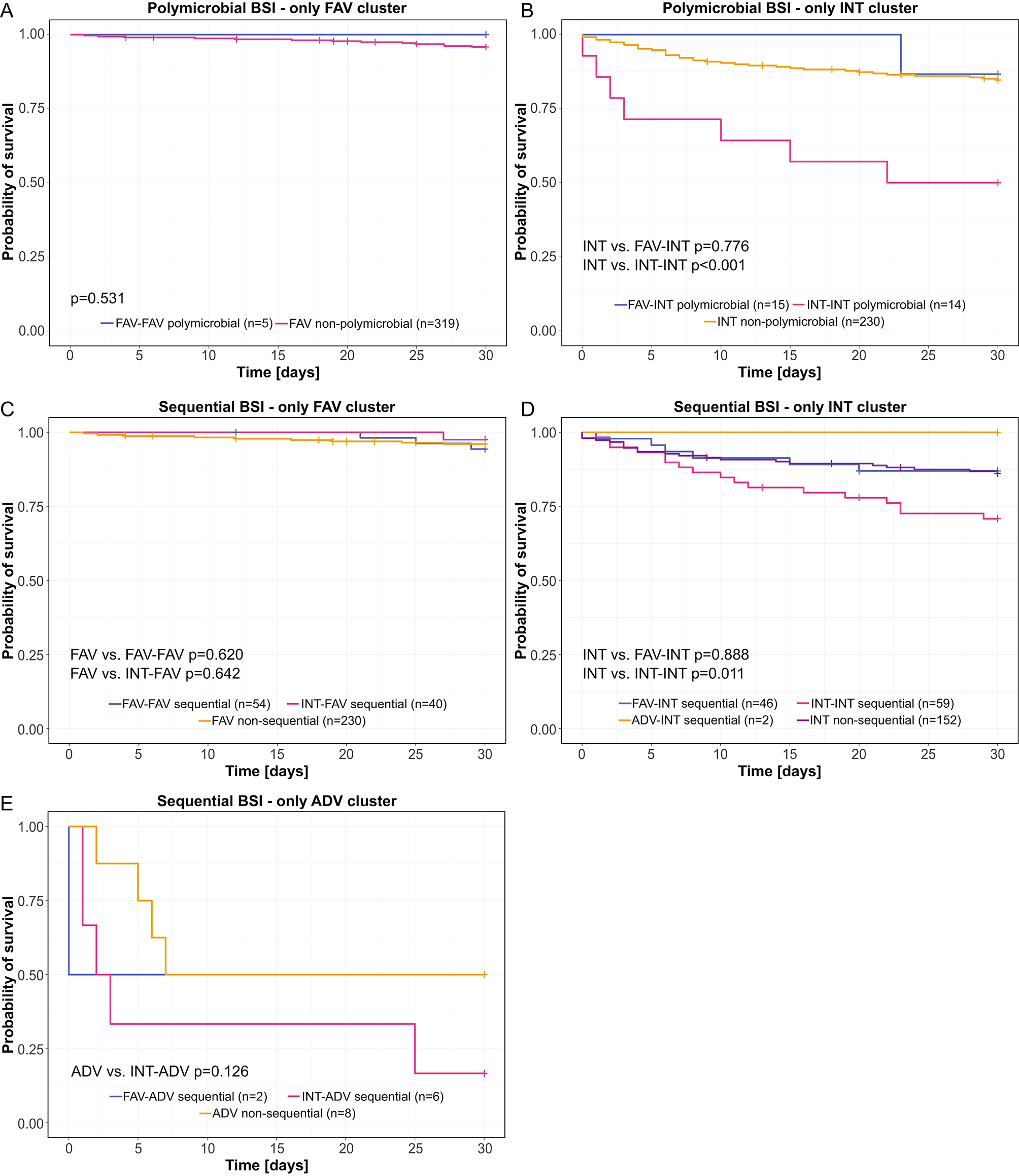

Supplement: Supplementary file 7 — Kaplan-Meier plots showing 30d OS for polymicrobial BSI episodes depending on the two highest parallel detected clusters and the corresponding non-polymicrobial BSI cluster stratified for the highest cluster present (A+B). Kaplan-Meier plots showing 30d OS for sequential BSI episodes depending on the highest previously detected cluster (first cluster) and the corresponding non-sequential BSI cluster stratified for the cluster of the current BSI episode (second cluster) (C-E). Groups with <5 BSI episodes were not included in the log rank test analysis and were only displayed descriptively. (PNG 493 kb). [file 277_2021_4541_Fig7_ESM.png]
