# Supplementary material for: Profiling of bacterial bloodstream infections in hematological and oncological patients based on a comparative survival analysis
Source: Ann Hematol. 2021 May 3;100(6):1593–602. doi: 10.1007/s00277-021-04541-9 (PMC8116230; doi:10.1007/s00277-021-04541-9)
Supplement: Supplementary file 9 — (DOCX 14 kb). [file 277_2021_4541_MOESM5_ESM.docx]

|  | **All patients (391)** | | **All BSI episodes (637)** | |
| --- | --- | --- | --- | --- |
| **MDS/AML/ALL** | 244 (62.4) | MDS: 9, AML: 187, ALL: 48 | 428 (67.2) | MDS: 18, AML: 327, ALL: 83 |
| **Lymphoma** | 100 (25.6) | CLL: 5, MM: 18, WM: 2, HL: 8, other: 67 | 141 (22.1) | CLL: 8, MM: 22, WM: 2, HL: 16, other: 93 |
| **MPN/MDS-MPN-Overlap** | 15 (3.8) | CML: 8, other MPN: 4, MDS-MPN-Overlap: 3 | 25 (3.9) | CML: 15, other MPN: 7, MDS-MPN-Overlap: 3 |
| **Non-malignant hematological diseases** | 14 (3.6) | VSAA: 6, AG: 2, Other: 6 | 22 (3.4) | VSAA: 9, AG: 2, Other: 12 |
| **Solid tumor** | 18 (4.6) | Lung cancer: 5, germ cell tumor: 4, osteosarcoma:2, other: 7 | 21 (3.3) | Lung cancer: 6, germ cell tumor: 4, osteosarcoma:3, other: 8 |

**Table S1:** Underlying hematological or oncological diseases for all patients and all BSI episodes, respectively. MDS, myelodysplastic syndrome; AML, acute myeloid leukemia; ALL, acute lymphoblastic leukemia; CLL, chronic lymphocytic leukemia; MM, multiple myeloma; WM, Waldenström's macroglobulinemia; HL, Hodgkin’s lymphoma; MPN, myeloproliferative disease; CML, chronic myeloid leukemia; VSAA, very severe aplastic anemia; AG, agranulocytosis.
