# Supplementary material for: Profiling of bacterial bloodstream infections in hematological and oncological patients based on a comparative survival analysis
Source: Ann Hematol. 2021 May 3;100(6):1593–602. doi: 10.1007/s00277-021-04541-9 (PMC8116230; doi:10.1007/s00277-021-04541-9)
Supplement: Supplementary file 10 — (DOCX 17 kb). [file 277_2021_4541_MOESM6_ESM.docx]

| **BSI group (number of episodes)** | **Genus (number of episodes)** | **Species (number of episodes)** |
| --- | --- | --- |
| CSC (common skin contaminants) (158) | *Staphylococcus* (157) | *S. epidermidis* (87), *S. haemolyticus* (23), coagulase-negative (unclassified, 47) |
|  | *Corynebacterium (1)* | *Corynebacterium jeikeium (1)* |
| EBAC (9) | *Enterobacter (9)* | *E. asburiae* (2), *E. cloacae* (4), *E. cloacae*-complex (unclassified, 3) |
| ECOC (83) | *Enterococcus (83)* | E. faecalis (21), faecium (59), gallinarum (3) |
| ESCH (121) | *Escherichia (121)* | E. coli (121) |
| KLEBS (16) | *Klebsiella (16)* | K. aerogenes (1), oxytoca (1), pneumoniae (14) |
| OTHEBAC (other *Enterobacterales*) (13) | *Citrobacter (1)* | C. koseri (1) |
|  | *Morganella (2)* | M. morganii (2) |
|  | *Pantoea (1)* | P. agglomerans (1) |
|  | *Proteus (3)* | P. mirabilis (3) |
|  | *Raoultella (1)* | R. ornithinolytica (1) |
|  | *Serratia (5)* | S. marescens (5) |
| PSEU (23) | *Pseudomonas (23)* | P. aeruginosa (22), alcaligenes (1) |
| RO_AN (rare organisms: anaerobic) (9) | *Actinomyces (1)* | A. neuii (1) |
|  | *Bacteroides (2)* | B. caccae (1), species (unclassified, 1) |
|  | *Blautia (1)* | R. gnavus (1) |
|  | *Fusobacterium (1)* | F. necrophorum (1) |
|  | *Leptotrichia (1)* | L. buccalis (1) |
|  | *Schaalia (1)* | S. odontolyticus (1) |
|  | *Veillonella (2)* | V. atypica (1), parvula (1) |
| RO_GN (rare organisms: gram-negative) (11) | *Achromobacter (1)* | A. xylosoxidans (1) |
|  | *Acetobacter (1)* | A. tropicalis (1) |
|  | *Acinetobacter (2)* | A. baumannii-complex (1), junii (1) |
|  | *Capnocytophaga (1)* | C. sputigena (1) |
|  | *Haematobacter (1)* | H. massilensis (1) |
|  | *Haemophilus (1)* | H. influenzae (1) |
|  | *Neisseria (1)* | N. flava (1) |
|  | *Sphingobacterium (1)* | S. thalpophilum (1) |
|  | *Sphingomonas (2)* | S. paucimobilis (2) |
| RO_GP (rare organisms: gram-positive) (15) | *Dermabacter (1)* | D. hominis (1) |
|  | *Gemella (1)* | G. haemolysans (1) |
|  | *Granulicatella (1)* | G. adiacens (1) |
|  | *Lactobacillus (5)* | L. curvatus (2), rhamnosus (3) |
|  | *Lactococcus (1)* | L. lactis (1) |
|  | *Listeria (2)* | L. monocytogenes (2) |
|  | *Okibacterium (1)* | O. fritillariae (1) |
|  | *Rothia (3)* | R. mucilaginosa (3) |
| STAPHA (16) | *Staphylococcus (16)* | S. aureus (14), aureus (MRSA) (2) |
| STENO (8) | *Stenotrophomonas (8)* | S. maltophilia (8) |
| STREP (32) | *Streptococcus (32)* | S. alactolyticus (1), gallolyticus (1), gordonii (1), mitis (1), oralis (3), parasanguis (1), pneumoniae (5), salivarius (1), sanguinis (3), viridans (unclassified, 15) |
| VRE (64) | *Enterococcus (VRE) (64)* | E. faecium (VRE) (64) |
| MDRGN (43) | *Enterobacter (MDRGN) (2)* | E. cloacae aeruginosa (MDRGN) (2) |
|  | *Escherichia (MDRGN) (23)* | E. coli (MDRGN) (23) |
|  | *Klebsiella (MDRGN) (12)* | K. oxytoca (MDRGN) (1), pneumoniae (MDRGN) (11) |
|  | *Pantoea (MDRGN) (1)* | P. agglomerans (MDRGN) (1) |
|  | *Pseudomonas (MDRGN) (5)* | P. aeruginosa (MDRGN) (5) |
| 4 MRGN (16) | *Acinetobacter (MDRGN+CR) (1)* | A. baumannii (MDRGN+CR) (1) |
|  | *Escherichia (MDRGN+CR) (1)* | E. coli (MDRGN+CR) (1) |
|  | *Klebsiella (MDRGN+CR) (3)* | K. pneumoniae (MDRGN+CR) (3) |
|  | *Pseudomonas (MDRGN+CR) (11)* | P. aeruginosa (MDRGN+CR) (11) |

**Table S2:** Detection number of different bacterial organisms with classification of bacterial species due to genus and BSI group.
