# Supplementary material for: Profiling of bacterial bloodstream infections in hematological and oncological patients based on a comparative survival analysis
Source: Ann Hematol. 2021 May 3;100(6):1593–602. doi: 10.1007/s00277-021-04541-9 (PMC8116230; doi:10.1007/s00277-021-04541-9)
Supplement: Supplementary file 11 — (DOCX 14 kb). [file 277_2021_4541_MOESM7_ESM.docx]

| **Bacterial organism group** | **Number at risk** | **30d OS, %** | **95% CI, %** |
| --- | --- | --- | --- |
| RO_AN | 9 | 100 | 100–100 |
| RO_GN | 11 | 100 | 100–100 |
| ESCH | 121 | 95.8 | 92.3–99.5 |
| CSC | 158 | 94.8 | 91.4–98.4 |
| STREP | 32 | 93.8 | 85.7–100 |
| KLEBS | 16 | 93.3 | 81.5–100 |
| MDRGN | 43 | 85.9 | 76.1–97.0 |
| ECOC | 83 | 85.5 | 78.2–93.4 |
| OTHEBAC | 13 | 84.6 | 67.1–100 |
| PSEU | 23 | 82.6 | 68.5–99.6 |
| RO_GP | 15 | 80.0 | 62.1–100 |
| VRE | 64 | 78.0 | 68.5–88.9 |
| STAPHA | 16 | 74.5 | 55.7–99.6 |
| EBAC | 9 | 66.7 | 42.0–100 |
| STENO | 8 | 62.5 | 36.5–100 |
| MDRGN+CR | 16 | 37.5 | 19.9–70.6 |

**Table S3:** Different bacterial organism groups with 30d OS in descending order. CI, confidence interval.
