# Supplementary material for: Profiling of bacterial bloodstream infections in hematological and oncological patients based on a comparative survival analysis
Source: Ann Hematol. 2021 May 3;100(6):1593–602. doi: 10.1007/s00277-021-04541-9 (PMC8116230; doi:10.1007/s00277-021-04541-9)
Supplement: Supplementary file 12 — (DOCX 13 kb). [file 277_2021_4541_MOESM8_ESM.docx]

| MDS/AML/ALL | 428 (67.2) | 228 (65.7) | 190 (69.3) | 10 (62.5) |
| --- | --- | --- | --- | --- |
| Lymphoma | 141 (22.1) | 83 (23.9) | 52 (19.0) | 6 (37.5) |
| MPN/MDS-MPN-Overlap | 25 (3.9) | 13 (3.7) | 12 (4.4) | 0 (0.0) |
| Non-malignant hematological diseases | 22 (3.4) | 11 (3.2) | 11 (4.0) | 0 (0.0) |
| Solid tumor | 21 (3.3) | 12 (3.5) | 9 (3.3) | 0 (0.0) |

**Table S4:** Comparison of underlying hematological or oncological diseases **between three BSI bacteria cluster (FAV, INT, ADV)**. MDS, myelodysplastic syndrome; AML, acute myeloid leukemia; ALL, acute lymphoblastic leukemia; MPN, myeloproliferative disease.
